# Spontaneous Tension Hemothorax in a Patient with Asbestosis

**DOI:** 10.5811/cpcem.2022.6.57031

**Published:** 2022-10-27

**Authors:** Toshinao Suzuki, Toshihiko Takada

**Affiliations:** * Teikyo University Chiba Medical Center, Interventional Radiology Center, Chiba, Japan; † Fukushima Medical University, Department of General Medicine, Shirakawa Satellite for Teaching And Research (STAR), Fukushima, Japan

**Keywords:** thoracic cavity, hemorrhage, shock

## Abstract

**Case Presentation:**

A 75-year-old man with a history of asbestosis presented to the emergency department with sudden-onset dyspnea and hemoptysis, triggered by coughing. The patient was hemodynamically unstable and in respiratory distress. Computed tomography revealed a massive hemothorax on the left side and compression of the descending thoracic aorta. He underwent emergency surgical exploration after decompression by chest tube insertion. The hemothorax was caused by tears in the pleural adhesions due to asbestosis and induced by coughing.

**Discussion:**

Spontaneous hemothorax is a rare subtype of hemothorax. There have been only a few case reports of spontaneous tension hemothorax. In addition to its typical findings, compression of the thoracic descending aorta was observed in our patient. We hypothesize that severely diminished pulmonary compliance contributed to the extremely high intrathoracic pressure, which led to this unusual finding.

## CASE PRESENTATION

A 75-year-old man with a history of asbestosis and cirrhosis with thrombocytopenia presented to the emergency department with sudden-onset dyspnea and hemoptysis triggered by coughing. He had a history of asbestos exposure during his tenure as an air-conditioning engineer 30 years prior. He had no trauma history and was not on anticoagulant or antiplatelet therapy. The patient was hemodynamically unstable and in respiratory distress with a blood pressure of 100/84 millimeters of mercury (mm Hg), pulse rate 116 beats per minute, respiratory rate 24 breaths per minute, and oxygen saturation level 90% on room air. Breath sounds were substantially decreased on the left side, and jugular vein distension was observed. Upon laboratory testing, the patient had a hemoglobin level of 12.4 grams per deciliter (g/dL) (reference range: 13.5–17.5 g/dL), platelet count of 42,000 per microliter (μL) (13,000–37,000/μL), prothrombin time of 16.5 seconds (10–13 seconds), and activated partial thromboplastin time of 26.3 seconds (20.0–38.0 seconds).

**Image 1:**
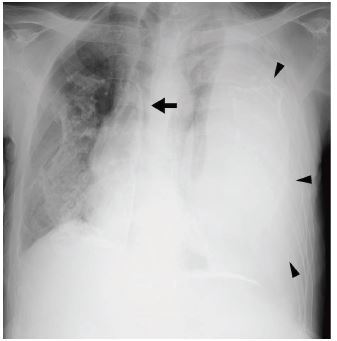
Chest radiograph (CXR) on the patient’s arrival to the emergency department. The CXR (anterior-posterior) before treatment shows bilateral, asbestos-calcified pleural plaques, a completely opaque left thorax (black arrowheads), and mediastinal shift toward the right (black arrow).

**Image 2:**
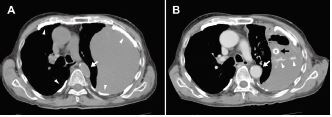
Computed tomography (CT) at the level of the tracheal bifurcation (A) before and (B) after chest drainage. (A) Plain CT at the level of the tracheal bifurcation shows mediastinal shift toward the right, compression of the descending thoracic aorta (arrow), massive left hemothorax, and multiple pleural plaques (arrowheads). (B) Contrast-enhanced CT after decompression of the left thorax shows improvement in the aortic compression (white arrow), a contrast blush indicating intrathoracic hemorrhage (arrowheads), and a drainage tube (black arrow) within the left-sided pleural effusion.

Chest radiography revealed bilateral pleural plaques, complete opacification of the left thorax, and mediastinal shift toward the right (Image 1). Computed tomography (CT) revealed massive hemothorax on the left and compression of the descending thoracic aorta (Image 2A). While undergoing CT, the patient’s systolic blood pressure dropped to 60 mm Hg. After draining 1,200 milliliters of blood by a chest tube insertion into the left thorax (Image 2B), his blood pressure increased to 98/62 mm Hg.

After transfusion of 20 units of platelets, surgical exploration was performed. Pleural adhesion tears and bleeding were detected. Histopathological examination of the torn parietal pleura with plaques revealed fibrinous pleuritis with massive fibrinosanguineous exudate. During postoperative mechanical ventilation, a severe reduction in pulmonary compliance was observed. After 26 days with delayed ventilator withdrawal and treatment of a urinary tract infection that developed during hospitalization, the patient fully recovered and was discharged. At one-year follow-up he was doing well without recurrence.

## DISCUSSION

Spontaneous hemothorax is a rare subtype of hemothorax, characterized by accumulation of blood within the pleural space, with no associated trauma or underlying cause. Conditions such as vascular malformations and neoplasms have been reported as potential causes.^1^ In our patient, hemothorax was caused by coughing-induced tears in the pleural adhesions due to asbestosis. This entity has been reported previously only in a patient with chronic obstructive pulmonary disease.^2^

The hemodynamic instability of our patient was attributed to obstructive shock due to tension hemothorax. Tension hemothorax is typically caused by major chest trauma or ruptured thoracic aortic aneurysm, and it rarely occurs spontaneously.^3^ In addition to the typical findings of tension hemothorax, such as opacification of the affected thorax and mediastinal shift,^4^ compression of the thoracic descending aorta was observed in our patient. Ideally, CT should not be performed before stabilization of the patient in such cases. In patients with asbestosis, asbestos fibers inhaled deep into the lungs get lodged in the tissues, eventually resulting in diffuse alveolar and interstitial fibrosis, leading to decreased lung compliance.^5^

**CPC-EM Capsule**

What do we already know about this clinical entity?

*Spontaneous hemothorax is a rare subtype of hemothorax with no associated trauma or underlying cause*.

What is the major impact of the image(s)?

*In addition to typical findings of tension hemothorax, computed tomography revealed compression of the descending thoracic aorta in a patient with asbestosis*.

How might this improve emergency medicine practice?

*In patients with decreased lung compliance, tension hemothorax could be caused not only by major trauma or ruptured aneurysm but also by spontaneous hemothorax*.
